# Biocontrol potential and comparative genomic analysis of two *Bacillus velezensis* strains against sclerotinosis in mulberry fruit

**DOI:** 10.3389/fmicb.2025.1587301

**Published:** 2025-06-25

**Authors:** Fangfang Peng, Xunlan Li, Zhaoxin Wei, Guohui Han

**Affiliations:** Fruit Research Institute, Chongqing Academy of Agricultural Sciences, Chongqing, China

**Keywords:** sclerotinosis, *Bacillus velezensis*, comparative genomes, biocontrol, mulberry

## Abstract

Mulberry trees (*Morus* spp.), a dual-purpose crop with nutritional and medicinal value, faces significant production constraints due to sclerotinosis. Two strains of *Bacillus velezensis* (JT-3 and JD-3) were isolated, demonstrating strong inhibitory effects against the sclerotinia pathogen (56.06% for JT-3 and 75.42% for JD-3). These strains effectively suppressed sclerotium formation and germination, achieving a complete inhibition rate. Field trials and growth promotion experiments showed that disease incidence in mulberry fruit could be reduced by up to 75.72%, with significant improvements observed in plant height, stem thickness, leaf number, root length, root weight, and above-ground biomass compared to the control group. Oxford Nanopore Technologies (ONT) sequencing showed the genome of JT-3 consists of a single circular chromosome with a size of 3.85 Mb, while the genome of JD-3 comprises a circular chromosome and a plasmid, with a total size of 4.12 Mb, including a plasmid of 35.7 kb. Both strains exhibit the capacity to secrete antifungal Carbohydrate-Active Enzymes (CAZymes) such as lysozyme and chitinase. Comparative genomics identified JD-3-specific enrichments in flagellar motility, hydrolase systems, antimicrobial defense clusters. Additionally, Both strains harbored 12 The biosynthetic gene clusters (BGCs), with JD-3 uniquely encoding plantazolicin synthesis. These findings provide foundational insights for developing *Bacillus*-based bioformulations while opening new avenues for the health management of mulberry fruit.

## 1 Introduction

Sclerotinosis is the most serious disease in the development of mulberry industry, which is a fungal disease and has the characteristics of rapid onset and wide range. Such diseases mainly caused by four pathogens, namely, *Ciboria shiraiana, Ciboria carunculoides, Scleromitrula shiraiana*, and *Sclerotinia sclerotiorum* (Ou et al., 2019). Chemical agents are routinely used to prevent sclerotinosis in mulberry fruits. The alternating application of thiophanate-methyl and putrescine has been reported to achieve control efficiencies exceeding 90%. However, high-performance liquid chromatography (HPLC) and gas chromatography (GC) analyses have revealed that pesticide residues in the fruit frequently surpass the established international safety limits (Li et al., 2018). These findings underscore the urgent necessity of tackling the irreversible deterioration of soil quality and the substantial health and environmental impacts linked to excessive dependence on chemical remedies. Moreover, the overuse of chemicals poses the threat of pathogen resistance (Dessalegn et al., 2013), potentially undermining the efficacy of current disease control strategies. Therefore, it is crucial to seek sustainable and environmentally friendly solutions to effectively solve this widespread disease in mulberry cultivation.

Plant endophytes, defined as fungi or bacteria inhabiting healthy plant tissues during specific or entire life stages, demonstrate vertical transmission capability to progeny while exerting significant positive impacts on host growth, health, development, and yield. These organisms constitute a critical reservoir for screening biocontrol agents (Hong and Park, 2016; Xiao et al., 2022; Drożdżyński et al., 2024). *Bacillus* spp., particularly notable for their biocontrol applications, are obligately aerobic or facultatively anaerobic, gram-positive, chemoorganotrophic bacteria (Yin et al., 2023). Their biocontrol efficacy stems from diverse secondary metabolites including enzymes, proteins, bacteriocins, polyketides, and lipopeptides - with polyketides and non-ribosomally synthesized lipopeptides (e.g., iturin, surfactin, fengycin) being predominant (Han et al., 2024; Kizhakkekalam et al., 2020). These compounds exhibit multifunctional bioactivities encompassing antimicrobial, antiviral, and phytostimulatory properties, holding substantial research value across industrial, agricultural, and biomedical sectors (Xu et al., 2018; Subramenium et al., 2018; Yuan et al., 2019; Dan et al., 2021; Petrović et al., 2023; Burragoni and Jeon, 2021).

*Bacillus velezensis*, a novel species within the *Bacillus* genus (Ye et al., 2018), has been extensively documented for its broad-spectrum antimicrobial activity, exceptional stress tolerance, and plant growth-promoting properties. Yang et al. (2023) isolated *B. velezensis* VJH504 from cucumber rhizosphere soil, demonstrating significant antagonistic activity against *Fusarium oxysporum f. sp. cucumerinum* FJH36 through Mycelial deformation and growth inhibition. The *B. velezensis* TSA32-1 exhibited protective effects on maize (*Zea mays*) and pepper (*Capsicum annuum*) seeds against phytopathogens including *Fusarium graminearum* and *Pythium ultimum*, exhibiting cellulase, lysozyme, and protease activities associated with the degradation of polymers in the cell wall of plant pathogens (Kim et al., 2022). Treatment with *B. velezensis* HMB26553 induced critical pathophysiological alterations in the phytopathogen *Rhizoctonia solani*, characterized by Mycelial structure changes, intracellular ROS hyperaccumulation, and a decrease in mitochondrial membrane potential. Concurrently, The *B. velezensis* HMB26553 exhibited indole-3-acetic acid (IAA) biosynthesis, siderophore production, extracellular enzyme secretion, biofilm formation, motility, and cotton growth-promoting capabilities (Su et al., 2023).

In this study, two strains of *B. velezensis* (JT-3 and JD-3) were isolated with significant inhibitory effects on the pathogen responsible for sclerotinosis from mulberry stem. To date, the biocontrol potential of *B. velezensis* against mulberry sclerotinosis remain scarcely reported. Antagonistic and growth-promoting assays were performed on these two dominant strains, and their biocontrol and growth-promoting potential were further investigated through whole-genome sequencing and functional annotation. Comparative genomic and secondary metabolite analyses with phylogenetically related strains provided molecular-level insights into their biocontrol mechanisms. This work aims to provide novel bacterial resources for the biological control of mulberry sclerotinosis and establish a practical foundation for field applications.

## 2 Materials and methods

### 2.1 Isolation, purification, and screening of biocontrol bacteria

#### 2.1.1 Sample collection and endophyte isolation

Healthy branches (cultivar Tu1) were collected at the Chongqing Academy of Agricultural Sciences experimental orchard (N29°27′38′′, E106°21′29′′) In June 2021, cut to appropriate lengths (approximately 2–3 cm), and rinsed under running water for 1 h. They were then transferred to a laminar flow cabinet, soaked in 75% ethanol for 2 min, and excess alcohol was burned off with forceps. The stems were then rolled on sterile Nutrient Agar (NA: 10.0 g Peptone, 3.0 g beef extract, 5.0 g NaCl, 18.0 g agar; pH 7.2) to agar to confirm absence of epiphytic microbiota. Subsequently, the sterilized stems were aseptically dissected into 5-mm transverse sections using sterile blade, placed on Potato Dextrose Agar (PDA: 200.0 g potato, 20.0 g glucose, 18.0 g agar; pH 7.2) and NA (25 ml per plate), and incubated at 25°C for 48 h. Emerging colonies were selected based on color, edge shape, and smoothness, and purified by triple streaking. Purified strains were stored at 4°C for later use.

#### 2.1.2 Primary screening of biocontrol bacteria

A dual-culture antagonism assay was conducted using *S. sclerotiorum* (Peng et al., 2024) as the target pathogen. The screening protocol comprised the following steps: Placement of 5-mm mycelial disc (48 h cultures) at the geometric center of 90 mm PDA plates. Using an inoculation loop, cross - inoculate four different strains 25 mm from the slice’s center. Culture at 25°C for 120 h. Antagonistic bacteria with inhibitory effects (compared to controls) on the pathogen were selected for later use.

#### 2.1.3 Secondary screening of biocontrol bacteria

Strains (single colonies) exhibiting antagonistic activity from primary screening were aseptically inoculated into 100 mL erlenmeyer flasks containing 20 mL Nutrient Broth (NB) and incubated at 180 rpm for 21 h at 33.0°C. Seed cultures (4% v/v inoculum) were transferred to 250 mL fresh NB medium in erlenmeyer flasks and cultivated under identical conditions until reaching OD_600_ = 1.70 ± 0.05. Antimicrobial activity was assessed using the agar well diffusion method: (1) A 5-mm mycelial disc from 48 h *S. sclerotiorum* cultures were centrally inoculated on PDA; (2) 5-mm wells were aseptically created 25 mm from the center using a diameter hole punch; (3) 20 μL bacterial fermentation broth was dispensed into test wells, with sterile NB as negative control. Triplicated plates were incubated at 25.0°C for 120 h. Inhibition percentage was calculated using digital image analysis (Image J 1.8.0). Sclerotial formation was quantified after 15 d.


Inhibition%=(1-B/tB)c×100.whereB=ccontrolcolonyarea(mm)2,B=ttreatedcolonyarea.

### 2.2 Morpho-physiological characterization of biocontrol bacteria

The target strains were streaked on NA medium and incubated at 33°C for 24 h to observe colony morphological characteristics. Single colonies were then gram-stained and spore-stained. Physiological and biochemical assays were performed using API 50CH system (bioMérieux, France) to assess carbon source utilization.

### 2.3 Resistance evaluation of of two *B. velezensis*

#### 2.3.1 Effect on mycelial morphology

Mycelial samples were collected from both the inhibition zone (experimental group) and undisturbed growth area (control group) as described in section “2.1.3. Secondary screening of biocontrol bacteria” These samples were stained with 0.4% Trypan Blue solution for 1 min and then examined under a light microscope to observe the morphological effects of the antagonistic strains on the pathogenic mycelium.

#### 2.3.2 Effects on sclerotia germination

Mature sclerotia (15-day-old) cultivated on PDA were collected and subjected to surface sterilization: (1) Immersion in 75% (v/v) ethanol for 2 min; (2) Three to five rinses with sterile distilled water, followed by; (3) Hydration in sterile water for 4–6 h at ambient temperature. After blot-drying with sterile filter paper, The sclerotia were subsequently embedded in sterile sand within 90-mm Petri dishes (10 sclerotia/dish). A volume of 200 μL of bacterial suspension (OD_600_ = 1.70 ± 0.05)was carefully pipetted onto the location of each sclerotium. Negative control groups received equivalent volumes of sterile NB. The experiment comprised three biological replicates. All petri dishes were placed in an incubator set at a constant temperature of 25°C for 1 week.

#### 2.3.3 Antifungal spectrum tests

The investigated fungal isolates were obtained from diseased mulberry fruits: *Periconia pseudobyssoides* (Accession: MN944517.1), *Epicoccum nigrum* (Accession: MN089646.1), *Fusarium acuminatum* (Accession: MT635295.1), *Didymella segeticola* (Accession: MT530451.1), *Alternaria alternata* (Accession: ON540391.1), and *Diaporthe vaccinii* (Accession: AB470842.1). The methodology for the tests was conducted in accordance with the protocol detailed in section “2.1.3. Secondary screening of biocontrol bacteria.”

### 2.4 Experiments on enzyme secretion and biofilm formation in biocontrol and plant growth-promoting systems

The functional characterization was performed using five specialized media systems: (1) Skim milk agar medium (0.1 g CaCl2, 5.0 g NaCl, 10.0 g peptone, 18.0 g agar; pH 7.2) for protease hydrolysis assessment, (2) Starch-enriched medium (10.0 g soluble starch, 10.0 g pancreatic casein digest, 5.0 g glucose, 5.0 g NaCl, 5.0 g beef extract, 18.0 g agar/L; pH 7.2) for amylolytic activity evaluation, (3) Cellulose-Congo red medium for cellulolytic capacity determination (Yang et al., 2023), and (4) Chrome Azurol S (CAS) agar for siderophore production analysis (Yang et al., 2023). Distinct clearance zones surrounding colonies served as positive indicators for each metabolic capability. Nitrogen fixation potential was examined using nitrogen-deficient basal medium (10.0 g C_6_H_14_O_6_, 0.2 g KH_2_PO_4_,0.2 g MgSO_4_⋅7H_2_O,0.2 g NaCl,0.2g CaSO_4_⋅2H_2_O,5.0 g CaCO_3_, 18.0 g agar; pH 7.2). Biofilm quantification employed a standardized 48 - well microtiter plate assay. Overnight cultures of activated strains in Tryptic Soy Broth (TSB: 17.0 g pancreatic peptone, 3.0 g soy papain hydrolysate, 2.5 g dipotassium hydrogen phosphate, 5.0 g sodium chloride, 2.5 g glucose; pH 7.3) and diluted appropriately (BM1, BM10, BM100, and BM500) in logarithmic phase (The logarithmic phase was determined according to the strain growth curve). Aliquots containing 1.5 mL MSgg medium supplemented with 10 μL inoculum were dispensed into wells (*n* = 5 replicates per treatment, PBS as negative control). Following 72 h static incubation at 30°C, culture medium were aspirated without disturbing the biofilm architecture. Triple PBS washes preceded air-drying under ambient conditions. Biofilms were subsequently fixed with 1% (w/v) crystal violet (15 min, 30°C), rigorously rinsed until eluent became colorless, then destained with 2 mL 33% glacial acetic acid (30 min). Absorbance measurements at 570 nm using a microplate spectrophotometer.

### 2.5 Growth promotion

Healthy, plump mulberry seeds with uniform size were germinated in substrate-filled trays (1:1 v/v sphagnum peat:lateritic soil). Seedlings with consistent growth were then transplanted into 15 cm × 25 cm pots containing growth medium (1:1 v/v sphagnum peat:lateritic soil). All pots were placed in a greenhouse. Root-irrigation trials were conducted on seedlings that had uniform growth stage (3–5 true leaves) and exhibited uniform growth characteristics. Briefly, bacterial suspensions (OD_600_ = 1.7 ± 0.05) were centrifuged at 6,000 rpm for 8 min. The supernatant was discarded, and the pellet was resuspended in an equal volume of sterile water to prepare the base inoculum (BM1). Serial dilutions (10-fold, 100-fold, 500-fold) yielded BM10, BM100, and BM500 treatments respectively. A volume of 20 mL of BM1, BM10, BM100, and BM500 was applied to the roots of the seedlings, respectively, with sterile water serving as the control (*n* = 10 replicates per group). Irrigation was performed every 48 h for a total of three times. Growth parameters were monitored weekly for 5 weeks: the number of leaves (fully expanded), plant height (soil surface to apical meristem), stem diameter (digital caliper at first internode). Terminal measurements at week 5 included: root weight, root length, and above-ground biomass.

### 2.6 Field test

To facilitate management and simulate natural field conditions, 2-year-old potted mulberry plants were placed in an orchard with diseased mulberry. Four different concentrations of bacterial suspensions (BM1, BM10, BM100, BM500) were prepared according to the method detailed in section “2.1.3. Secondary screening of biocontrol bacteria,” with sterile water serving as the control. The bacterial suspensions were sprayed onto the leaves during the flowering period of the mulberry trees, starting from the tree canopy and continuing until the leaves began to drip. This spraying method was performed weekly for three consecutive weeks, with each concentration applied to 20 pots. After fruiting, the fruits were bagged to monitor disease incidence.


Diseaseincidence(%)=(infectedfruits/totalfruits)×100

### 2.7 Extraction of DNA, library construction, and whole-genome sequencing

The genomic DNA of biocontrol bacteria was extracted using the HiPure Bacterial DNA kit (Magen, Guangzhou, China) according to the manufacturer’s instructions, which was validated through tripartite analysis using a NanoDrop 2000 spectrophotometer (Thermo Scientific) for purity (A260/A280), QubitTM3 fluorometer (Invitrogen) for concentration quantification, and 0.35% agarose gel electrophoresis (Tanon) for integrity assessment. For nanopore sequencing library preparation, 2 μg of high-quality DNA was mechanically fragmented using G-TUBE devices (Covaris) under optimized centrifugal conditions. DNA fragment repair and terminal modification were systematically performed using NEBNext FFPE DNA Repair Mix (New England Biolabs, Ipswich, MA) and NEBNext Ultra II End Repair/dA-Tailing Module (New England Biolabs), ligation of barcode sequences and sequencing adapters using the PCR-free Barcoding Expansion Kit 1–12 Oxford Nanopore Technologies (ONT). Libraries meeting stringent quality criteria (QubitTM3 fluorometric quantification > 8 ng/ul) were sequenced on a PromethION48 platform (ONT).

### 2.8 Genome assembly, prediction and annotation

The raw sequencing reads were filtered and assembled using Canu v1.5 (Koren et al., 2017). The assembly results were then corrected using Racon v3.4.3 software with third-generation reads. The Circlator v1.5.5 software was employed for circularization and adjustment of start sites. Ribosomal RNA (rRNA) genes were predicted using Infernal v1.1.3 (Nawrocki and Eddy, 2013), and transfer RNA (tRNA) genes were identified using tRNAscan-SE v2.0 (Chan and Lowe, 2019). CRISPR structures were predicted using CRT v1.2 (Bland et al., 2007). Genomic islands were predicted using IslandPath-DIMOB v0.2 (Bertelli and Brinkman, 2018), and prophages were identified using PhiSpy v2.3 (Akhter et al., 2012). Gene prediction was performed using Prodigal v2.6.3 (Hyatt et al., 2010), and were annotated by comparing them against various databases, including the Non-Redundant (Nr) database, UniProt, Clusters of Orthologous Groups (COG), and the Kyoto Encyclopedia of Genes and Genomes (KEGG). Additionally, protein sequences of the genes were aligned with Hidden Markov Models (HMMs) from the CAZy database (Zheng et al., 2023).

### 2.9 Phylogenomic and comparative genomic analysis

Orthologous single-copy genes were identified using the software OrthoFinder^1^, and a phylogenetic tree was constructed for 25 selected strains of *Bacillus* (including JD-3 and JT-3) using concatenated multiple gene sequences.

Average nucleotide identities (ANIs) and *in silico* DNA-DNA hybridization (DDH) values were determined using the OrthoANIu algorithm (Richter and Rosselló-Móra, 2009) and the Genome-to-Genome Distance Calculator (GGDC) (Meier-Kolthoff et al., 2013). Comparative genomic analyses were conducted using MAUVE software (Ye et al., 2022).

### 2.10 Secondary metabolism gene cluster analysis and validation

The biosynthetic gene clusters (BGCs) were identified and analyzed in the bacterial genome sequence using the antiSMASH v7.0 software (Blin et al., 2023). To validate genes associated with secondary metabolite biosynthesis, a random selection of functional genes related to secondary metabolite synthesis from the identified BGCs, including *PKSI* (polyketide synthase I), *NRPS* (non-ribosomal peptide synthetase), *Sfp*, *srfC*, *ItuD*, and *FenD* (Gond et al., 2015) were subjected to PCR amplification (The primers used are detailed in Supplementary Table 1).

### 2.11 Statistical analyses

The Statistical data from antibacterial assays and plant growth promotion experiments were conducted using GraphPad Prism 9. One-way ANOVA with Tukey’s *post hoc* test was applied for multiple comparisons, with statistical significance threshold set at *p* < 0.05 (two-tailed). The statistical analysis of CAZymes gene families was performed using Microsoft Excel 2020. The BGCs distribution was analyzed using Cloud Platform of GENE DUNOVO^2^.

## 3 Results

### 3.1 Morphological and physiological biochemical characteristics

**FIGURE 1:**
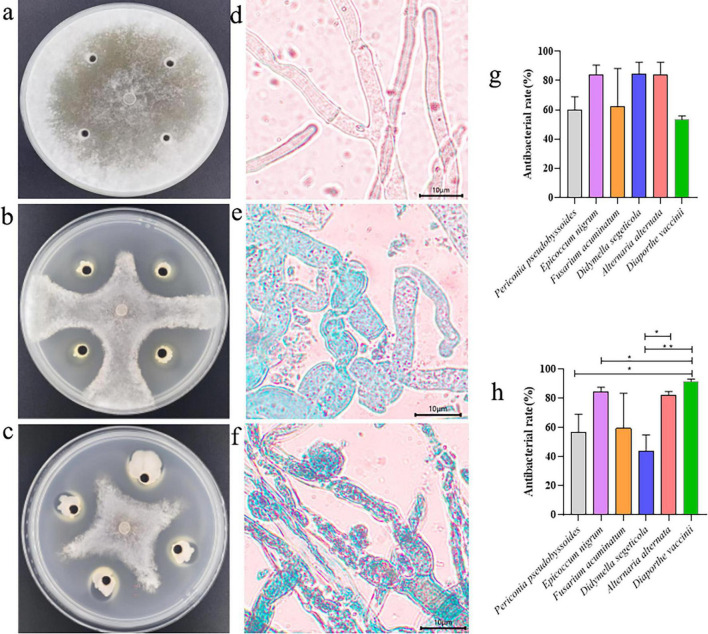
Inhibition of pathogenic fungi by fermentation broth of JT-3 and JD-3. **(a)** Control. **(b)** Inhibitory effect of JT-3 on *S. sclerotiorum*. **(c)** Inhibitory effect of JD-3 on *S. sclerotiorum*. **(d)** Mycelial characteristics of *S. sclerotiorum* in the control group. **(e)** Effect of JT-3 on the growth of *S. sclerotiorum* mycelium. **(f)** Effect of JD-3 on the growth of *S. sclerotiorum* mycelium. **(g)** Inhibitory effect of JT-3 on other pathogenic fungi. **(h)** Inhibitory effect of JD-3 on other pathogenic fungi. *p < 0.05 and **p < 0.01 indicate statistical significance.

Twenty-one morphologically distinct bacterial isolates were purified from mulberry stems. Primary antagonistic screening against *S. sclerotiorum* obtained nine strains with inhibitory activity. Subsequent secondary screening prioritized two superior candidates, JT-3 and JD-3, exhibiting 56.06% (Figure 1b and Table 1) and 75.42% (Figure 1c and Table 1) mycelial growth suppression, respectively. JT-3 and JD-3 strains were cultured on NA at 33°C for 24 h. Both exhibited milky-white colonies with irregular margins and opaque morphology. Specifically, JT-3 displayed slightly wrinkled surfaces without viscoelasticity (Supplementary Figure 1). Gram staining revealed Gram-positive, rod-shaped cells existing singly or in chains, accompanied by endospore formation (Supplementary Figure 1). Physiological profiling showed no significant metabolic differences except in the utilization of D-Mannose, N-Acetylglucosamine, and D-Melibiose (Supplementary Table 2). Combined with morphological traits, both strains were provisionally identified as members of the *Bacillus* genus.

**TABLE 1:** Inhibitory effects of initial screening strains.

Strains	Colony area (mm^2^)	Inhibition (%)
JD-1	47.82 ± 1.41 bc	53.14 ± 1.56 bc
JD-2	46.90 ± 1.20 bc	52.11 ± 1.33 bc
JD-3	67.88 ± 2.17 a	75.42 ± 2.41 a
JC-1	43.40 ± 1.54 cd	48.22 ± 1.71 cd
JC-8	40.45 ± 1.24 d	44.94 ± 1.38 d
JC-9	48.43 ± 1.20 bc	53.82 ± 2.22 bc
JT-2	49.44 ± 0.79 b	54.94 ± 0.88 b
JT-3	50.43 ± 1.65 b	56.03 ± 1.84 b
JT-8	45.15 ± 1.37 bcd	50.16 ± 1.52 bcd

Different lowercase letters indicate significant differences at the 0.05 level.

### 3.2 Effect of two *B. velezensis* fermentation solutions on the morphology of pathogenic fungi

In the dual-culture assays, a very obvious inhibitory zone was observed at the interface between the target biocontrol strains and the pathogenic fungus. Microscopic analysis of confrontation zones revealed distinct morphological alterations in pathogenic hyphae, characterized by cellular enlargement, distortion, and localized wall lysis (Figure 1). Vital staining confirmed membrane integrity loss (Figures 1e, f), whereas the control mycelia appeared robust, smooth, and intact (Figure 1d). Of particular significance was the complete inhibition (100%) of sclerotial formation during 15-day co-cultivation. Subsequent sclerotial germination assays in sterile soil demonstrated total inhibition (100%) upon treatment with fermentation broth (Supplementary Figure 2). Furthermore, antifungal spectrum tests indicated that both strains possessed broad-spectrum antifungal activity against other fungal pathogens (Figures 1g, h).

### 3.3 Detection of biological control-related enzymes, plant growth-promoting substances, and biofilm formation

**FIGURE 2:**
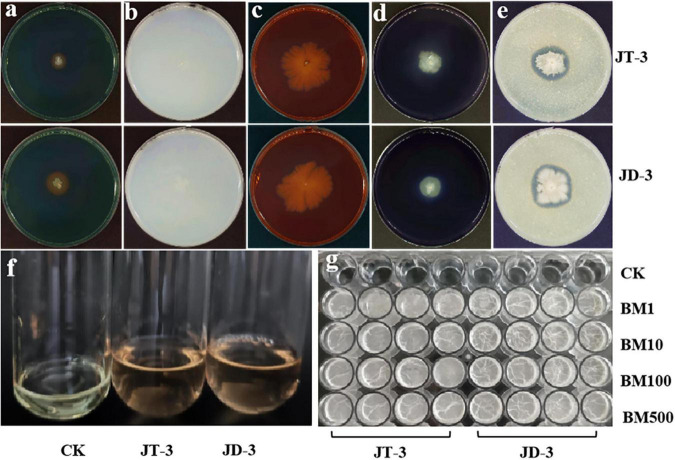
Antifungal and plant growth-promoting characteristics and biofilm formation. **(a)** Production of siderophore. **(b)** Nitrogen fixation. **(c)** Production of cellulasesproteases. **(d)** Production of amylases. **(e)** Production of proteases. **(f)** Production of IAA. **(g)** Biofilm detection.

The plate assays results revealed that both strains produced protease and amylase, enzymes siderophores, and IAA. The cellulose-Congo red assays showed limited cellulolytic activity, while nitrogen-free medium supported vigorous growth, suggesting that they possess nitrogen-fixing capabilities (Figures 2a–f). Biofilm formation capacity was quantified using crystal violet retention assays. Strain JD-3 exhibited significantly higher biofilm biomass (A570 = 3.76 ± 0.05) compared to JT-3 (A570 = 1.32 ± 0.28) (Figure 2g), indicating that JD-3 has a significantly stronger biofilm-forming ability than JT-3, which is advantageous for the strain’s environmental adaptability and could contribute to improved colonization and biocontrol efficacy within the host plant.

### 3.4 Effects of two *B. velezensis* on seedling growth

**FIGURE 3:**
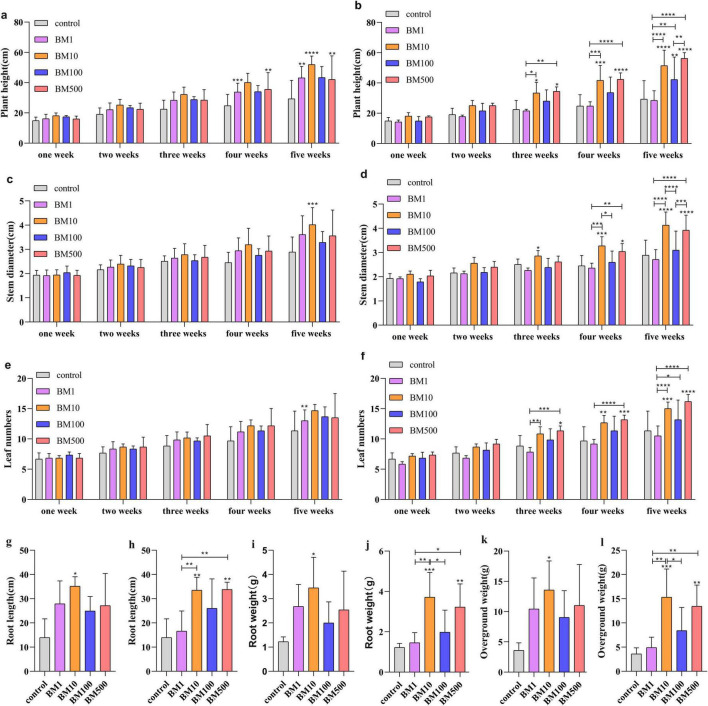
Effects of two *B. velezensis* on seedling growth. **(a)** Effect of JT-3 on plant height. **(b)** Effect of JD-3 on plant height. **(c)** Effect of JT-3 on stem diameter. **(d)** Effect of JD-3 on stem diameter. **(e)** Effect of JT-3 on the leaf number. **(f)** Effect of JD-3 on the leaf number. **(g)** Effect of JT-3 on root length. **(h)** Effect of JD-3 on root length. **(i)** Effect of JT-3 on root weight. **(j)** Effect of JD-3 on root weight. **(k)** Effect of JT-3 on the aboveground weight. **(l)** Effect of JD-3 on the aboveground weight. *p < 0.05, **p < 0.01, and ***p < 0.001 indicate statistical significance.

Continuous 5-week monitoring of biocontrol-treated seedlings showed that as time progressed, significant differences emerged between the treated groups and the control. The BM10 and BM500 treatment exhibited pronounced growth advantages, particularly in plant height, stem diameter, leaf number, root weight, root length, and shoot fresh weight (Figures 3a–l and Supplementary Figure 3). At the fifth week evaluation, JT-3 (BM10) treatment increased plant height (Figure 3a), stem diameter (Figure 3c), root length (Figure 3g), root weight (Figure 3i), and root weight (Figure 3k) by 77.26, 38.96, 152.46, 182.26, and 278.79%, respectively, compared to controls. JD-3 (BM10) enhanced corresponding parameters (Figures 3b, d, f, h, j, l) with 75.38, 42.91, 32.35, 140.84, 204.09, and 327.53% increments. JD-3 (BM500) demonstrated 91.87, 35.76, 42.65, 155.09, 163.98, and 275.40% improvements in key growth metrics.

### 3.5 Evaluation of field trial

**FIGURE 4:**
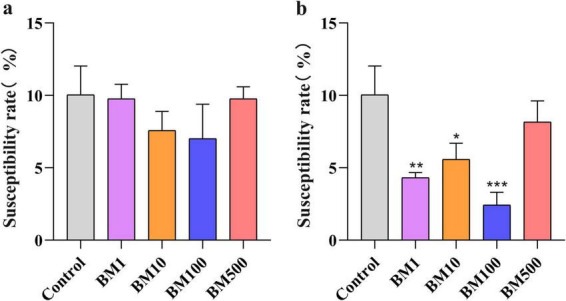
Effect of biocontrol bacteria on the incidence of sclerotinosis in the field (%). **(a)** Susceptibility rate under JT-3 treatment. **(b)** Susceptibility rate under JD-3 treatment. *p < 0.05, **p < 0.01, and ***p < 0.001 indicate statistical significance.

Field trials revealed distinct Sclerotinia disease incidence rates: 7.02%–9.80% for JT-3 and 2.44%–8.17% for JD-3, with the latter demonstrating significantly lower pathogenicity (BM1, BM10, BM100; *p* < 0.05) compared to controls (Figure 4b). Both strains exhibited superior disease suppression efficacy at the BM100, whereas BM500 showed reduced inhibitory activity (Figure 4). Relative to untreated controls, JT-3 and JD-3 applications reduced disease incidence by 30.14 and 75.72%, respectively, confirming JD-3’s superior field performance. Optimal pathogen suppression was achieved with BM100, highlighting its practical utility for agricultural deployment.

### 3.6 Genome sequencing, assembly, and annotation of two *B. velezensis*

**FIGURE 5:**
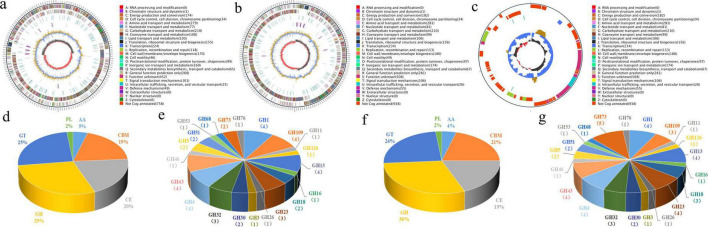
Genomic circular maps and CAZymes analysis of JT-3 and JD-3. **(a)** Genomic circular maps of JT-3. **(b)** Genomic circular maps of JD-3. **(c)** Circular map of the plasmid in JD-3. The outermost circle is the genome size mark (5 kb scales). The second and the third circles are the genes on the positive and negative chains of the genome, respectively, and different colors represent different functional classifications of COG. The fourth circle has the repeated sequence. The fifth circle has tRNA and rRNA, (blue is tRNA, purple is rRNA). The sixth circle shows the GC content, the light yellow part indicates that the GC content of this region is higher than the average GC content of the genome, the higher the peak value, the greater the difference between the average GC content, and the blue part indicates that the GC content of this region is lower than the average GC content of the genome. The innermost ring shows the GC-skew, with dark gray representing regions with more G than C and red representing regions with more C than G. **(d)** CAZymes profiling of JT-3. **(e)** GH family analysis of JT-3. **(f)** CAZymes profiling of JD-3. **(g)** GH family analysis of JD-3.

The JT-3 features a 3.85 Mb circular chromosome (GC = 46.73%; N50 = 2.1 Mb), containing 3,677 protein-coding genes, 27 rRNA, 86 tRNA, 36 non-coding RNAs, 4 CRISPR arrays, 4 genomic islands, 2 prophages, and 12 gene clusters (Figure 5a). Functional annotation revealed 2,577, 2,140, 3,663 genes mapped to GO, KEGG, NR databases (Table 2), respectively. JD-3 harbors a 4.09 Mb circular chromosome (46.48% GC) and a 35.7 kb plasmid, encoding 4,018 protein-coding genes, 27 rRNA, 87 tRNA, 36 non-coding RNAs, 4 CRISPR arrays, 4 genomic islands, 3 prophages, and 12 gene clusters (Figures 5b, c). Functional annotation revealed 3,080, 2,963, 2,184 genes mapped to GO, KEGG, NR databases (Table 2).

**TABLE 2:** Database annotation statistics.

Database	Number	
	JD-3	JT-3
eggNOG annotation	3,080	2,919
GO annotation	2,963	2,577
Kegg annotation	2,184	2,140
Nr annotation	4,002	3,663
Pfam annotation	3,478	3,285
Swissprot annotation	2,865	2,785
TrEMBL annotation	3,986	3,656
All annotated	4,011	3,670

### 3.7 CAZymes analysis

The CAZymes profiling serves as a vital indicator of the application potential of microbial strains. For strain JT-3, a total of 155 genes were annotated to the CAZy, which included 45 glycoside hydrolases (GH), 39 glycosyltransferases (GT), 3 polysaccharide lyases (PLs), 31 carbohydrate esterases (CE), 7 auxiliary activities (AA), and 30 carbohydrate-binding modules (CBMs) (Figure 5d). For strain JD-3, a total of 164 genes were annotated to the CAZy database, comprising 49 GH, 40 GT, 3 PLs, 31 CE, 7 AA, and 34 CBMs (Figure 5f). Notably, GH families were significantly enriched with antifungal-related domains, including genes encoding lysozyme (GH73), β-glucanase (GH1), chitinase (GH23/GH18), cellulase (GH5/GH43), α-glucosidase (GH4), and α-amylase (GH13), genetically confirming their antifungal enzyme secretion potential (Figures 5e, g).

### 3.8 Phylogenetic tree construction and genome comparison

**FIGURE 6:**
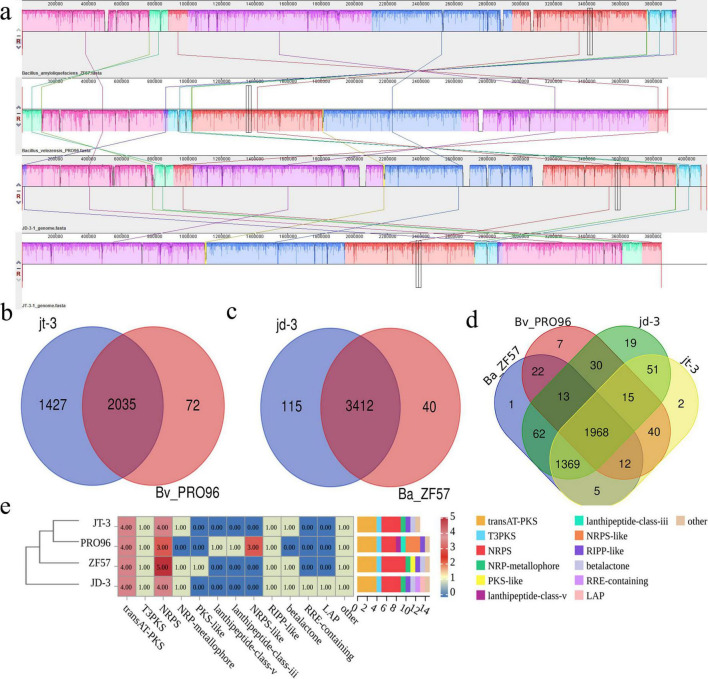
Comparative genomic analysis of JT-3 and JD-3. **(a)** Collinearity analysis. **(b)** Distribution of shared and unique orthologous clusters among JT-3 and PRO96. **(c)** Distribution of shared and unique orthologous clusters among JD-3 and ZF57. **(d)** Distribution of shared and unique orthologous clusters among the four strains. **(e)** Distribution of biosynthetic gene clusters (BGCs) of four strains.

Phylogenetic analysis (Supplementary Figure 4a) positioned strain JT-3 within the *B. velezensis* clade alongside PRO96, while JD-3 clustered with *B. amyloliquefaciens* ZF57. To validate these phylogenetic relationships, we computed ANI and DDH values following established species delineation thresholds (ANI > 96%; DDH ≥ 70%) (Jiang et al., 2022). The JT-3/PRO96 pair exhibited ANI and DDH values of 98.8% and 98.9%, respectively, surpassing species demarcation criteria. Interestingly, JD-3/ZF57 demonstrated values of 97.7% (ANI) and 94.5% (DDH), exceeding the proposed thresholds (Supplementary Figure 4b),In combination with the NR data, JD-3 was classified as a member of *B. velezensis* (Supplementary Figure 4c). Comparative genomic analysis using Mauve revealed high synteny between JT-3/PRO96 and JD-3/ZF57 (Figure 6a), with collinear blocks indicating conserved genome architecture. Structural variations were limited to localized rearrangements, devoid of detectable insertions, deletions, or inversions. Venn diagram quantification (Figures 6b–d) identified 2,035 shared orthologous clusters between JT-3 and PRO96, with 1,427 and 72 gene families, respectively. The JD-3/ZF57 pair shared 3,412 core clusters, while harboring 115 and 40 unique gene families, respectively. Functional annotation of unique gene families through GO enrichment analysis revealed notable adaptations in JD-3, and were associated with cilium-mediated motility, defense response mechanisms, hydrolase activity (Supplementary Figure 5), suggesting specific evolutionary trajectories.

### 3.9 Gene cluster analysis

Bioinformatics prediction of BGCs using antiSMASH revealed the secondary metabolic potential of both strains. JT-3 and JD-3 each harbored 12 putative BGCs, including non-ribosomal peptide synthetases (NRPS), trans-acyltransferase polyketide synthases (transAT-PKS), and type III polyketide synthases (T3PKS) as predominant types (Figure 6e, Supplementary Table 3). Comparative analysis with four strains genomes identified six conserved BGCs encoding bacillaene, fengycin, difficidin, bacilysin, surfactin, and macrolactinH. Notably, JD-3 uniquely possessed plantazolicin-related clusters (Table 3 and Supplementary Table 3). These findings collectively demonstrate the strains’ biosynthetic arsenal aligns with biocontrol bacteria database, while also maintaining unique metabolic signatures. The clustering results of BGCs were inconsistent with the phylogenetic tree (Supplementary Figure 2a), indirectly indicating that BGCs of biocontrol strains cannot be used as the sole basis for constructing phylogenetic trees. Six genes related to secondary metabolite biosynthesis were randomly selected for amplification, confirming the genomic presence of key biosynthetic genes (*PKSI, NRPS, Sfp, ItuD, Srfc*) in both strains (Supplementary Figure 4d).

**TABLE 3:** Comparative prediction of secondary metabolites.

Name	Bacillaene	Fengycin	Difficidin	Bacilli- bactin	Bacilysin	Surfactin	AndalusicinA/ Andalusicin B	Amylocy- clicin	Macrolacin H	bacillothia zol A-N	Plantazolicin
JT-3											
PRO96											
ZF57											
JD-3											

Green highlighting indicates the presence of the corresponding substance.

## 4 Discussion

Two *B. velezensis* strains (JT-3 and JD-3) with potent inhibitory activity against *S. sclerotiorum* were isolated from mulberry stems in this study. These strains demonstrated significant suppression of sclerotial formation and germination, enriching the biocontrol resources in mulberry and providing a novel strategy for its management. Field trials are planned to evaluate foliar and soil applications at optimized concentrations. The safety profile of *B. velezensis* is well-documented. Notably, this species holds Qualified Presumption of Safety (QPS) status from the European Food Safety Authority (EFSA) (Koutsoumanis et al., 2020). Strain TS5 enhanced intestinal digestive enzymes, hepatic antioxidant capacity, and modulated gut microbiota in murine models (Chen et al., 2023). Dietary supplementation with *B. velezensis* in European seabass (Dicentrarchus labrax) improved immune responses and survival rates (Monzón-Atienza et al., 2022). Genomic analysis of JT-3/JD-3 revealed no homologs to major pore-forming enterotoxins (*Hbl*, *Nhe*, *CytK*) through Virulence Factor Database (VFDB) alignment (Dimopoulou et al., 2021). While putative virulence-associated genes were identified (Supplementary Table 4), none corresponded to established toxin categories.

Endophytic bacteria, as integral components of the phytomicrobiome, enhance nutrient bioavailability, improve stress resilience (both abiotic and biotic), and stimulate secondary metabolite biosynthesis (Zhao et al., 2024). Carbohydrates serve dual roles in these symbioses - functioning as primary carbon sources while mediating host-microbe adhesion and establishing infection barriers (Wardman et al., 2022). The enzymatic depolymerization of carbohydrates requires coordinated action of CAZymes classified under GHs (Cantarel et al., 2009). Genomic analyses of *Bacillus* spp. reveal conserved CAZymes arsenals including chitinases, amylases, chitosanases, lysozymes, and β-glucosidases, which can disrupt fungal cell structures and dissolve cell walls, thereby exhibiting antifungal, pathogen-inhibiting, antibiotic-activating, and nutrient-absorbing effects (Douka et al., 2024; Huang et al., 2023; Wang et al., 2024; Xie et al., 2023; Yang et al., 2023). The CAZymes analysis of JD-3 and JT-3 is consistent with previous studies, indicating that both strains have the potential to degrade and utilize fungal polymers as a nutrient source, which is also proven by the experimental results of active substance secretion of the target strains.

The identification and analysis of BGCs provide an understanding of the various ways biocontrol bacteria exert their antagonistic effects (Xiao et al., 2022). Three principal lipopeptide families - surfactin, iturin (a iturin-family variant), and fengycin - were identified as non-ribosomal peptide synthetase (NRPS)-derived metabolites. These lipopeptides are known for their direct action on fungal biofilms, causing osmotic imbalances and cell death through the formation of voids (Falardeau et al., 2013). Their antifungal effects are so significant that they surpass those of fungicides like carbendazim (Krishnan et al., 2019; Park et al., 2019). Difficidin (Arguelles-Arias et al., 2009) and bacilysin (Nannan et al., 2021) both exhibit broad-spectrum antimicrobial activities, suggesting substantial potential for further development. MacrolactinH displayed dose-dependent inhibition of phytopathogenic fungal development, including conidial germination, germ tube elongation, and mycelial growth (Ni et al., 2023). Bacillibactin is directly linked to biocontrol and reduces the risk of *Cephalosporium maydis* infection in maize (Ghazy and El-Nahrawy, 2021), and siderophores can inhibit pathogens directly and indirectly induce host defenses (Hatzinikolaou and Skandalis, 2021). While bacillaene and butirosin demonstrated pharmaceutically relevant antibacterial properties (Erega et al., 2021), their direct biocorrelation with plant disease suppression remains unsubstantiated, warranting further functional genomics investigation. antiSMASH analysis confirmed conserved BGCs for these metabolites across both strains. Notably, JD-3 produces the unique substance plantazolicin, which is a thiopeptide exhibiting Gram-positive antibacterial activity (Zhang et al., 2022) and efficacy against Xanthomonas campestris (Liu et al., 2013). Additionally, three orphan gene clusters lacking database homologs in JD-3 and JT-3 (Supplementary Table 4) suggest potential novel antimicrobial compounds requiring heterologous expression validation.

Endophytic bacteria are increasingly utilized as plant growth promoters through rhizosphere modification and phytohormone biosynthesis (Zhao et al., 2024). These microbes enhance nutrient acquisition efficiency (N, P, K) and synthesize auxins like IAA (Wang et al., 2024; Teixeira et al., 2021; Yuan et al., 2022; Toral et al., 2020). For instance, the endophytic strain B.L.Ns.14 significantly promotes the growth of unstressed Arabidopsis and mitigates the harmful effects of salt stress on Arabidopsis seedlings, while also positively influencing tomato agronomic traits (Douka et al., 2024). In this study, we analyzed the plant - growth - promoting genes in JD-3 and JT-3, focusing on genes related to phosphorus metabolism, nitrogen fixation, siderophore production, IAA synthesis, and biofilm formation (Supplementary Table 5). *In vitro* tests showed that these strains can produce siderophores and IAA. Pot trials also demonstrated their significant ability to enhance the growth of mulberry seedlings.

Effective colonization capacity, a critical evaluative metric for biocontrol agents, is defined by the establishment of stable biofilms onplant surfaces (Liu et al., 2020). This colonization process constitutes an essential prerequisite for subsequent pathogen antagonism and phytostimulation functionalities (Philippot et al., 2013). In *Bacillus* spp., environmental cues activate histidine kinases (*KinA/B/C/D*) that initiate autophosphorylation cascades. The phosphotransfer relay (*Spo0F → Spo0B → Spo0A*) culminates in Spo0A phosphorylation (*Spo0A*∼*P*∼), which upregulates *Sin*I/AbbA expression while suppressing *SinR/AbrB* transcriptional repressors. Concurrently, the *YwcC-SlrA-SlrR* pathway facilitates *SlrR-SinR* heterodimer formation, derepressing biofilm matrix operons (*yqxM-sipW-tasA, epsA-O*) and downregulating autolysins and motility genes (Fujita and Losick, 2005; Gao, 2015), ultimately promoting biofilm formation. Genomic analysis identified JD-3-specific gene clusters enriched in flagellum-dependent motility, hydrolase activity, and defense response systems-traits mechanistically linked to chemotaxis-mediated plant colonization (Teixeira et al., 2021; Fujinami et al., 2009). Based on the above, we speculate that the antagonistic and growth-promoting abilities of JD-3 and JT-3 are closely linked to their biofilm-forming capabilities. Their potential as biocontrol agents and plant growth promoters is significant, warranting further field trials.

## Data Availability

The complete genome sequences of Bacillus strains JD-3 and JT-3 have been deposited in GenBank under the following accessions: JD-3: Chromosome CP174519, plasmid CP174520; JT-3: Chromosome CP178562.

## Footnotes

1
https://github.com/davidemms/OrthoFinder

2
https://www.omicshare.com/tools/
