# Supplementary material for: Biocontrol potential and comparative genomic analysis of two Bacillus velezensis strains against sclerotinosis in mulberry fruit
Source: Front Microbiol. 2025 Jun 25;16:1587301. doi: 10.3389/fmicb.2025.1587301 (PMC12237919; doi:10.3389/fmicb.2025.1587301)
Supplement: Supplementary file 1 [file Data_Sheet_1.pdf]

Supplementary Material

Supplementary Figures

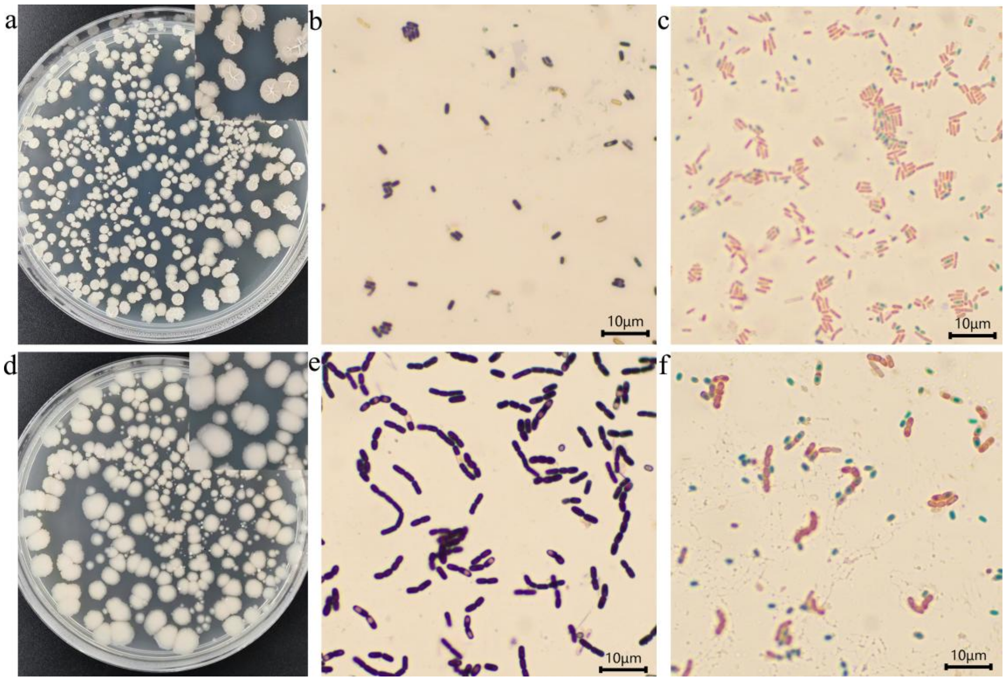

Supplementary Figure 1. Colony characteristics, gram staining, and spore staining of JT-3 and JD-3. a, Colony Characteristics of JT-3; b, Gram Staining of JT-3; c, Spore Staining of JT-3; d, Colony Characteristics of JD-3; b, Gram Staining of JD-3; c, Spore Staining of JD-3.

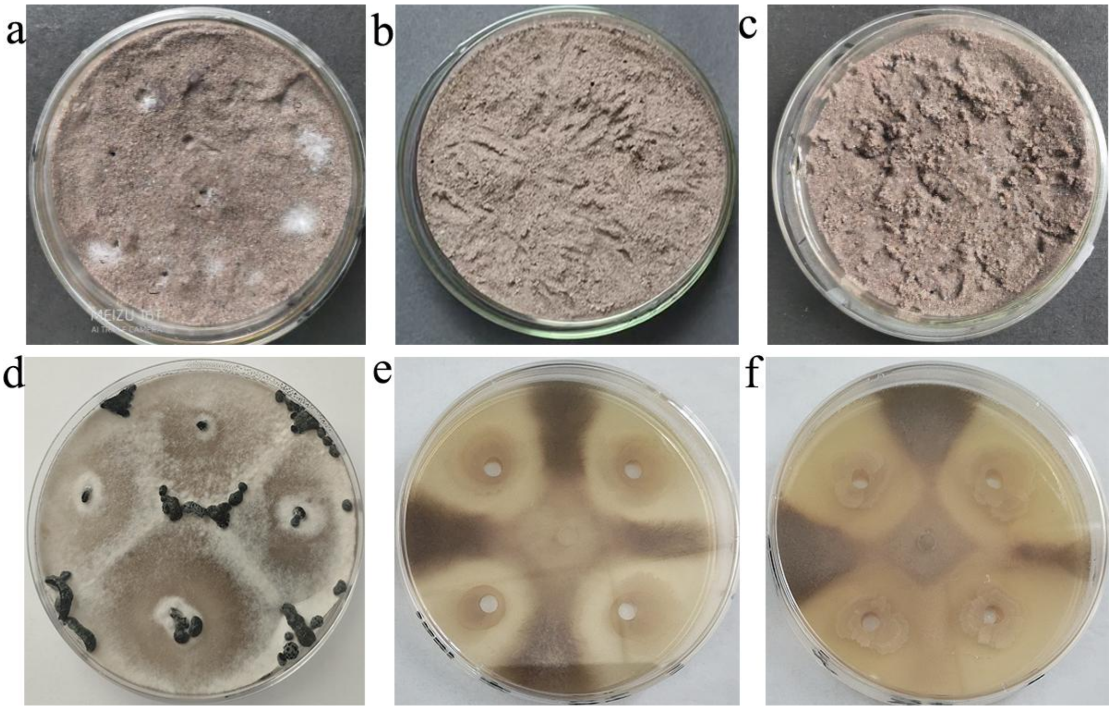

Supplementary Figure 2. Effect of the fermentation broth on the germination and formation of sclerotia. a, Sclerotium germination in the control; b, Inhibitory effect of JT-3 on germination of sclerotia; c, Inhibitory effect of JD-3 on germination of sclerotia; d, Sclerotium formation in the control; e, Effects of JT-3 on sclerotium formation; f, Effects of JD-3 on sclerotium formation.

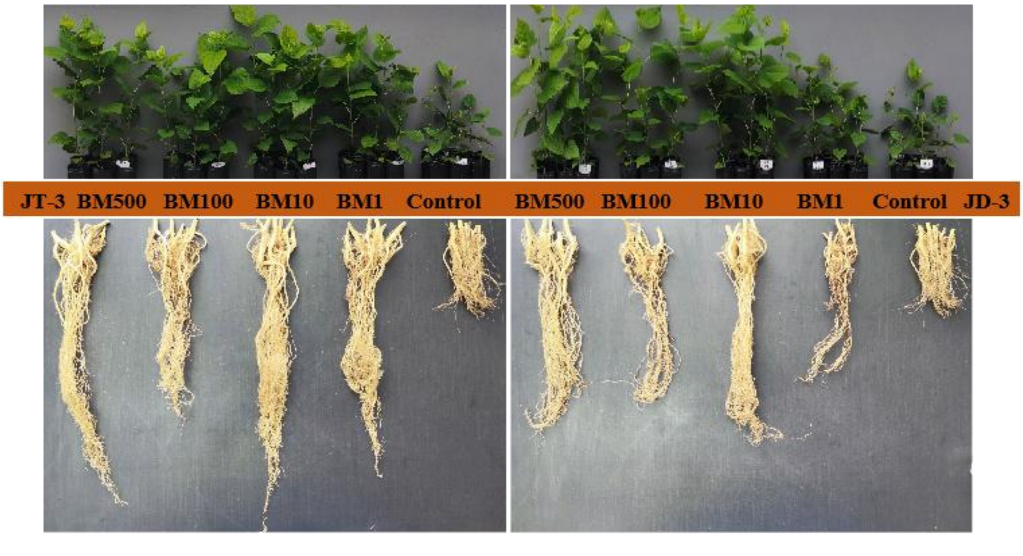

Supplementary Figure 3. Growth of mulberry seedlings under JT-3 and JD-3 treatments.

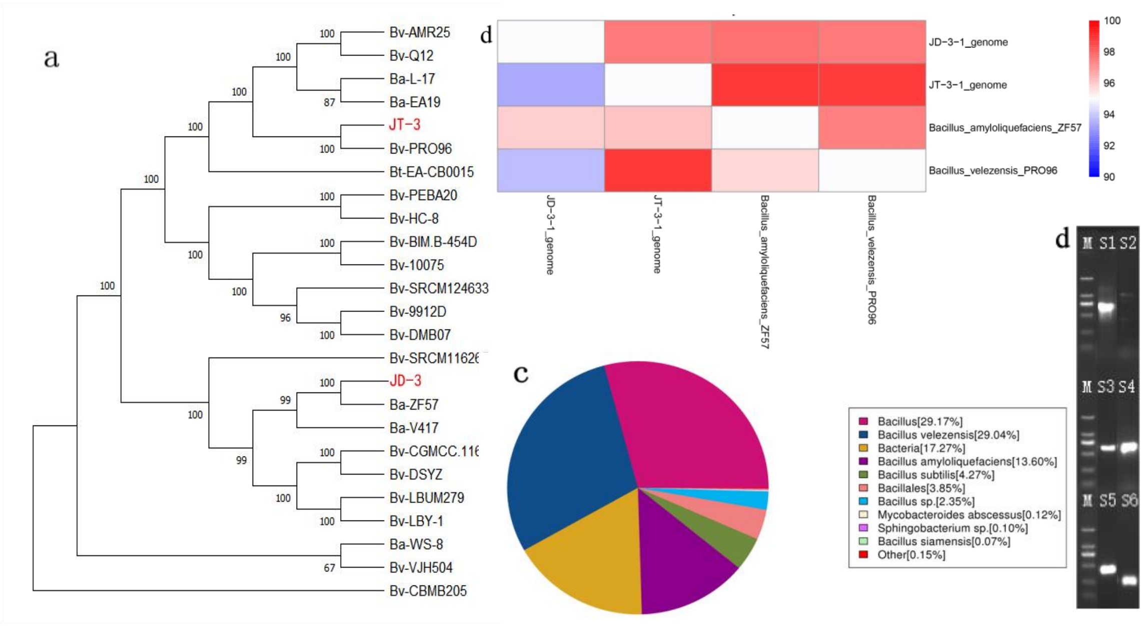

Supplementary Figure 4. a， phylogenetic trees of JT-3 and JD-3; b, ANI and DDI heatmaps of JT-3 and JD-3; c,The species distribution map of sequences matched to the Nr database; d, PCR validation of some bacteriostatic substances.

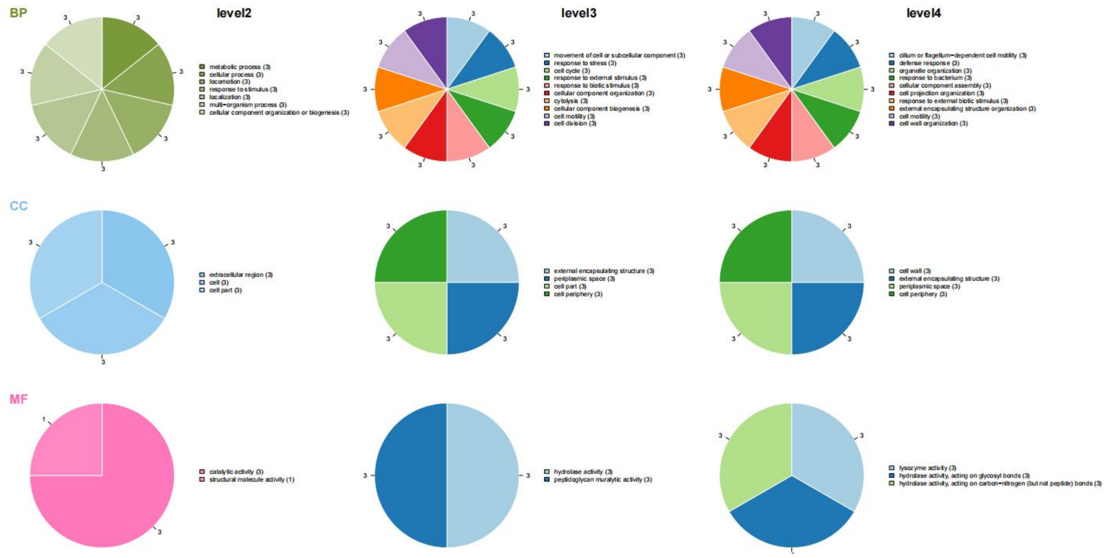

Supplementary Figure 5. GO analysis of JD-3 specific gene family.

Supplementary Tables

Supplementary Table 1 The sequence of gene primers for the synthesis of bacteriostatic substances.

No	Antibiotic	Target gene	Primer	Primer sequence(5'to3')	Base number
S1	PKSI	PKSI	KSF	GCGATGGATCCNCAGCAGCG	20
			KSR	GTGCCGGTNCCGTGNGYYTC	20
S2	NRPS	NRPS	NRPSF	GCNGGYGGYGCNTAYGTNCC	20
			NRPSR	CCNCGDATYTTNACYTG	17
S3	Surfactin	Sfp	SfpF	ATGAAGATTTACGGAATTTA	20
			SfpR	TTATAAAAGCTCTTCGTACG	20
S4	Iturin A	ItuD	ItuD1F	GATGCGATCTCCTTGGATGT	20
			ItuD1R	ATCGTCATGTGCTGCTTGAG	20
S5	Surfactin	Srfc	Sur3F	ACAGTATGGAGGCATGGTC	19
			Sur3R	TTCCGCCACTTTTTCAGTTT	20
S6	Fengycin	FenB	fenDF	GGCCCGTTCTCTAAATCCAT	20
			fenDR	GTCATGCTGACGAGAGCAAA	20

Supplementary Table 2 Physiological and biochemical characteristics of JT-3 and JD-3.

Substance	JD-3	JT-3	Substance	JD-3	JT-3	Substance	JD-3	JT-3
Glycerol	+	+	Mannitol	+	+	D-Ricinose	+W	+W
D-Fructose	+	+	N-Acetylglucosamine	-	+W	D-Turanose	-	-
β-Methyl-D-Xyloside	-	-	Salicin	+W	+W	D-Fucose	-	-
D-Mannose	+	-	D-Melibiose	+W	-	Potassium Gluconate	-	-
Inositol	+W	+	D-Rhamnose	-	-	L-Arabinose	+	+
α-Methyl-D-Glucoside	+W	+	Xylitol	-	-	Adonitol	-	-
Aesculin	+	+	D-Tagatose	-	-	D-Fructose	+	+
D-Lactose	+	+	L-Arabitol	-	-	Euonymus Alcohol	-	-
Inulin	-	-	D-Arabinose	-	-	α-Methyl-D-Mannoside	-	-
2-Keto-Gluconate	-	-	L-Xylose	-	-	Arbutin	+W	+W
D-Lyxose	-	-	D-Glucose	+	+	D-Maltose	+	+
D-Arabitol	-	-	L-Rhamnose	-	-	D-Trehalose	+W	+
5-Keto-Gluconate	-	-	Sorbose	+	+	D-Digitalose	-	-
Mannitol	-	-	Amygdalin	+W	+W	L-Fucose	-	-
D-Xylose	+W	+W	D-Cellobiose	+	+			
D-Galactose	-	-	D-Sucrose	+W	+			
L-Sorbose	-	-						

Note: "+" indicates a positive result, "+W" indicates a weakly positive result, and "-" indicates a negative result.

Supplementary Table 3 Comparison of gene clusters and core genes involved in antibiotic biosynthesis in JT-3 and JD-3 as well as *Bacillus velezensis* PRO96, *Bacillus amyloliquefaciens* ZF57.

Strains		bacillaene	fengycin	difficidin	bacillibactin	bacilysin	surfactin	unkown	andalusicin A/andalusicin B	amylocyclicin	macrolacin H	bacillothiazol A-N	plantazolicin
JT-3	Antibiotic	√	√	√	√	√	√				√		
	Type	transAT-PKS,T3PKS,NRPS	NRPS,transAT-PKS,	transAT-PKS	NRP-metallophore,NRPS,RIPP-like	other	NRPS	terpene,T3PKS, lanthipeptide-class-v			transAT-PKS		
	Core biosynthetic genes	GE000702,GE000703,GE000704,GE000706,GE000709,GE000710,GE000711,GE000712,GE000713,	GE000814, GE000815, GE000816,GE000817,GE000833,GE000836,GE000840,GE000841,GE000842,GE000843,GE000844,	GE001199, GE001200, GE001201,GE001202,GE001203,GE001204,GE001205,GE001210,	GE001890, GE001893, GE001894, GE001911,	GE003076, GE003077, GE003078,	GE003493, GE003494,	GE000081, GE000901, GE001016, GE003493, GE003494			GE000444, GE000445, GE000446, GE000447, GE000448, GE000449, GE000450		
PRO96	Antibiotic	√	√	√		√	√		√	√	√		
	Type	transAT-PKS,NRPS-like,NRPS,T3PKS,	NRPS-like,NRPS, transAT-PKS,	transAT-PKS		other	NRPS, NRPS-like	T3PKS, Lanthipeptide-class-v	Lanthipeptide-class-iii	RIPP-like	transAT-PKS		
	Core biosynthetic genes	P5660_15550, P5660_15570, P5660_15575, P5660_15580, P5660_15585, P5660_15595, P5660_15610, P5660_15615, P5660_15625, P5660_15635, P5660_15655, P5660_15665	P5660_14705, P5660_14755, P5660_14785, P5660_14790, P5660_14800, P5660_14920, P5660_14925, P5660_14940, P5660_14955, P5660_14965, P5660_14985	P5660_12595, P5660_12635, P5660_12645, P5660_12650, P5660_12665, P5660_12670		P5660_05995	P5660_02530, P5660_02545, P5660_02550, P5660_02555, P5660_02570	P5660_13690, P5660_00330, P5660_00335	P5660_03240	P5660_08880	P5660_17005, P5660_17015, P5660_17020, P5660_17035, P5660_17040, P5660_17045, P5660_17065, P5660_17070, P5660_17080, P5660_17100		
ZF57	Antibiotic	√	√	√	√	√	√				√	√	
	Type	transAT-PKS,T3PKS,NRPS	NRPS,transAT-PKS,betalactone	transAT-PKS	NRP-metallophore, NRPS,RiPP-like	other	NRPS	terpene, T3PKS			transAT-PKS	NRPS	
	Core	LOZ87_RS08760,	LOZ87_RS09385,	LOZ87_RS11230,	LOZ87_RS14	LOZ87_RS1	LOZ87_RS01	LOZ87_RS056			LOZ87_RS07460,	LOZ87_RS14	

	biosynthetic genes	LOZ87_RS08765, LOZ87_RS08770, LOZ87_RS08780, LOZ87_RS08795, LOZ87_RS08800, LOZ87_RS08805, LOZ87_RS08810, LOZ87_RS08815	LOZ87_RS09390, LOZ87_RS09395, LOZ87_RS09400, LOZ87_RS09480, LOZ87_RS09495, LOZ87_RS09515, LOZ87_RS09520, LOZ87_RS09525, LOZ87_RS09530, LOZ87_RS09535	LOZ87_RS11235, LOZ87_RS11240, LOZ87_RS11245, LOZ87_RS11250, LOZ87_RS11255, LOZ87_RS11260, LOZ87_RS11285	810, LOZ87_RS14 825, LOZ87_RS14 830, LOZ87_RS14 920	7910	795, LOZ87_RS01 800, LOZ87_RS01 810, LOZ87_RS01 820	30, LOZ87_RS098 35, LOZ87_RS103 20		LOZ87_RS07465, LOZ87_RS07470, LOZ87_RS07475, LOZ87_RS07480, LOZ87_RS07485, LOZ87_RS07490	015	
JD-3	Antibiotic	√	√	√	√	√	√			√		√
	Type	transAT-PKS, T3PKS, NRPS	NRPS, transAT-PKS, betalactone	transAT-PKS	NRP-metallop hore, NRPS, RiPP-like	other	NRPS	terpene, T3PKS		transAT-PKS		RRE-containing, LAP
	Core biosynthetic genes	GE001685, GE001686, GE001687, GE001689, GE001692, GE001693, GE001694, GE001695, GE001696	GE001808, GE001809, GE001810, GE001811, GE001827, GE001830, GE001834, GE001835, GE001836, GE001837，GE001838	GE002249, GE002250, GE002251, GE002252, GE002253, GE002254, GE002255, GE002260	GE003097, GE003100, GE003101, GE003118	GE003685	GE000345, GE000346，GE000347	GE001060, GE001954, GE002066		GE001429, GE001430, GE001431, GE001432, GE001433, GE001434, GE001435		GE000713, GE000714, GE000715

Supplementary Table 4　Virulence factor prediction in JT-3 and JD-3.

Strains	VFDB_gene_name
JT-3	fbpC) iron(III，isdG，esaA，lplA1，chuY，pchD，cylA，bauB，basA，cheV3，flmH，sipA，lbtC，phzH，pchR，bopD，bplA，tagAB-5，tlpB，pvdH，cylI，fbpC) iron(III，hitC) iron(III，hitC) iron(III，fbpC) iron(III，bapC，flmH，cylA) ABC (ATP-binding cassette，fes，farB，galE，hddA，mgtC，rhlB，chuV，bopD，ami，gtrB，mucD，farB，fbpC) iron(III，iap/cwhA，hitC) iron(III，cba，lgtF，cylA，fepC，sfaX，fliA，pilS，pilS，sfaX，motD，motA，clpE，msrA/B(pilB)，mgtB，bopD，acfB，gnd，fleS/flrB，Cj1437c，cheV，cylG，ptxR，bplB，fbpC) iron(III，sipA，bauE，pilS，allB，clbG，wcbR，clbI，wcbR，wcbR，wcbR，clbI，wcbR，clbP，pdgA，aur，algU，kdtB，vpdC，mucD，wecA，cpsG，fliA，fliA，fbpC) iron(III，lspA，allB，cysC，fbpA，mgtB，wbkC，pppA，ppkA，clbG，cylG，acpXL，flhF，flhF，flhB，fimB，clpE，flgB，flgC，fliE，fliF，fliG，cdsL，cdsN，flgD，flgG，fliL，fliM，fliN，cheY，fliP，fliQ，fliR，flhB，flhA，flhF，fleN，cheB，cheA，cheW-2，cheD，fliA，cpsA，cpsB，mucP，CBU_1434，pdgA，essC，cylG，clbP，wcbT，clbB，clbB，clbB，pvdL，wcbR，wcbR，pvdI，wcbR，cylA，eccA3，wcbR，cheB-2，msrA/B(pilB)，cylG，bopD，phtA，phoR，phoP，pvdI，pvdI，pvdD，clbG，cylG，wcbT，pvdH，hasC，entE，clbF，pvdI，pvdL，pvdI，pvdI，pvdL，fimE，dep/capD，allS，clbB，allS，cylG，flmH，exsA，iap/cwhA，lap，sodB，sodCI，iap/cwhA，iroB，lpxE，pvdH，kfiC，cap8E，pseB，msrA/B(pilB)，msrA/B(pilB)，fleQ/flrC，AHA_3493，per，lpsB/lpcC，Cj1437c，amoA，cheR-3，ebp，algU，phoR，bfmR，msrA/B(pilB)，sipA，lirB，fliA，fimE，flmH，clbI，wcbR，clbI，wcbR，wcbR，clbI，wcbR，cylG，entE，clbB，allS，gnd，fbpC) iron(III，pebA，lafK，icl) Isocitrate lyase Icl (isocitrase，clbF，clbD，cheB，recN，vpdC，ideR，lplA1，cylF，pilE，mshG) MSHA (mannose-sensitive hemagglutinin，xcpR，hlyA，hitC) iron(III，hitC) iron(III，sodB，CBU_2076，fliA，CBU_1594，lpg2936，chuW，pce，pdgA，farB，coxH2，icaR，fbpC) iron(III，pebA，relA) Probable GTP pyrophosphokinase RelA (ATP:GTP 3'-pyrophosphotransferase) (PPGPP synthetase I) ((P)PPGPP synthetase，iap/cwhA，basG，flhG，pilD，pvdH，vasH/clpV，clpE，orfM，sfaX，csgD，pchD，algR，algZ，phoR，phoP，cylG，entE，pvdN，icaR，pchD，motB，pomA2，bopD，kdsA，essC，wzt，bopD，hitC) iron(III，fleS/flrB，bfmR，fbpC) iron(III，hitC) iron(III，ricA，phzH，fbpC) iron(III，napA，luxS，entE，pchA，lap，bslA/yuaB，aut，bplA，acfB，acfB，acfB，acfB，hlyA，lap，lap，Cj1437c，rhlA，Cj1437c，fleS/flrB，lbtB，fleS/flrB，fleR/flrC，chuV，bprB，phzD1，entA，esaA，essC，essB，esaB，esxA，mbtH，pvdD，entB，pchD，amoA，entA，iroE，wzt，cylG，adsA，allB，lbtC，allC，fbpC) iron(III，pvdN，rtxB，IlpA，fbpC) iron(III，clbF，clbD，sfaX，farB，allS，fepC，cylG，napA，mucD，bfmR，bfmS，bprB，fepC，chuU，shuT，flmH，CBU_1789，phoR，phoP，fepC，shuU，fepD，bauB，bprB，cylA) ABC (ATP-binding cassette，mgtB，mgtB，bplA，hitC) iron(III，allS，cylG，bfmS，bfmR，cylA) ABC (ATP-binding cassette，fbpC) iron(III，fbpC) iron(III，per，rffG，bplF，tviB，scpA，lbtC，bopD，allS，bopD，rpoN，per，neuD，cap8M，kfoC，cpsE，cps4H，wbcG，wbbO，cap8D，cap8B，cps4C，bopD，clpP，allS，lbtC，iap/cwhA，phoR，bfmR，msbA，wcsT，ybtQ，farB，bslA/yuaB，fbpC) iron(III，fliS，fliD，flaB，fliW，flgL，lfgK，bprB，cps4A，wecA，kfiC，algD，waaG，cap8M，cpsA，cps4I，hasC，cpsJ，cpsK，cpsC，cpsD，rfaE，cpsC，lytB，kfiC，cpsA，iap/cwhA，capA，capC，capB，bopD，rfaE，fbpC) iron(III，ptxR，dep/capD，ptxR，algD，cap8C，cap8B，cap8A，fabZ，flgG，flgF，farB，phzD1，plcD，ureB，ureA，ureA，yscN，yscN，wzb，escN，phoP，plcD，msbA，plcD，prfA，cylR2，hitC) iron(III，allS，cylR2，flmH，Cj1437c，cylG，lbtC，lbtC，bslA/yuaB，cap8F，rffG，rffG，hasC，flmD，neuB，bplF，Cj1434c，kfoC，scpA，gtcA，allS，mycP3，lgtC，relA) Probable GTP pyrophosphokinase RelA (ATP:GTP 3'-pyrophosphotransferase) (PPGPP synthetase I) ((P)PPGPP synthetase，pvdI，algI，fepD，fepG，bauB，katA，bsh，hlyB，ybtQ，icaB) N-deacetylase, involved in polysaccharide intercellular adhesin(PIA，fbpC) iron(III，galE，katA，fepB，fbpC) iron(III，fleS/flrB，bfmR，bplA，cylG，bprB，pvdH，lafK，bauE，phoR，algW，phoR，bfmR，lsgC，cps4H，lbtC，bopD，exsA，AHA_1389，entB，wcbM，coxK1，prsA2，ybtS，phzE1，clpC，bsc1，bplB，algU，rtxB，hitC) iron(III，flhG，pdgA，pvdH，fepG，fepD，fepB，pchR，clbP，algU，algC，fepC，bprB，cylA) ABC (ATP-binding cassette，hpt，pvcC，fleS/flrB，farB，icaR，cylG，lmb，bauE，mntB，lbtC，fbpC) iron(III，farB，chpE，phtA，pvdL，pvdL，pvdI，ybtT，clbA，fbpC) iron(III，allS，iraB，pebA，fbpC) iron(III，fbpC) iron(III，bfmR，phoR，bauD，bauC，bauE，bauB，farB，pvdH，cylG，fabZ，allR，pchD，flmH，bplI，icaA) N-acetylglucosaminyltransferase, involved in polysaccharide intercellular adhesin(PIA，fleS/flrB，fleR/flrC，cpsJ，clbF，lafS，lipF，sfaX，coxH2，clbB，pilH，Cj1417c，ccmA，lbtC，cylI，iroB，clbP，clbB，coxH2，barB，htpB，clbB，hldE，oatA，algU，wcbR，algU，phzH，wbkC，mgtC，clbL，icaR，acrB，cylA) ABC (ATP-binding cassette，cylB) ABC (ATP-binding cassette，CBU_1566，isdG，fbpB) iron(III，fbpB) iron(III，fbpC) iron(III，ddhA，ddhB，Cj1135，tlpA，flmH，lgtF，chuV，shuV，wcbR，fleS/flrB，cheY，adsA，bopD，wzb，brkB，pdgA，cylA) ABC (ATP-binding cassette，hitC) iron(III，fleQ，cyaB，msbA，icaC) intercellular adhesion protein C, involved in polysaccharide intercellular adhesin(PIA，farB，phzF1，sprE，gtrB，cylG，cyaB，pvdH，fbpC) iron(III，hitC) iron(III，katA，fbpC) iron(III，cylR2，farA，farB，bauE，hitC) iron(III，IlpA，adsA，srtC，pvdH，yhxB/manB，rcsB，iap/cwhA，iap/cwhA，allS，flmH，algU，hlyA，Cj1437c，

	cylI，hlyA，pdgA，msbA，msbA，chuW，basC，cylA) ABC (ATP-binding cassette
JD-3	VFDB_gene_name，lbtC，bopD，chpD，AHA_1389，entB，wcbM，coxK1，prsA2，ybtS，phzE1，clpC，bsc1，bplB，algU，rtxB，fbpC) iron(III，flhG，pdgA，pvdH，fes，fepG，fepD，fepB，pchR，clbP，algU，algC，fepC，bprB，cylA) ABC (ATP-binding cassette，hpt，pvcC，fleS/flrB，chpA，farB，icaR，cylG，lmb，bauE，mntB，lbtC，fbpC) iron(III，farB，chpE，phtA，phtA，pvdL，pvdL，pvdI，ybtT，clbA，fbpC) iron(III，allS，iraB，pebA，fbpC) iron(III，fbpC) iron(III，bfmR，phoR，bauD，bauC，bauE，bauB，farB，pvdH，cylG，fabZ，allR，pchD，flmH，bplI，pgaC，fleS/flrB，fleR/flrC，cpsJ，clbF，lafS，hldD，flmH，clbB，clbB，pilH，Cj1417c，ccmA，lbtC，cylI，iroB，clbP，clbB，coxH2，barB，htpB，clbB，hldE，oatA，algU，wcbR，algU，phzH，wbkC，mgtC，clbL，icaR，mtrD，cylA) ABC (ATP-binding cassette，cylB) ABC (ATP-binding cassette，CBU_1566，isdG，fbpB) iron(III，fbpB) iron(III，fbpC) iron(III，sfaX，phzS，cylA) ABC (ATP-binding cassette，llsD，llsB，ddhA，ddhB，Cj1135，tlpA，flmH，lgtF，chuV，shuV，wcbR，phoR，algR，fss1，srtE，fleS/flrB，cheY，adsA，bopD，wzb，brkB，pdgA，fleQ，cyaB，msbA，icaC) intercellular adhesion protein C, involved in polysaccharide intercellular adhesin(PIA，farB，phzF1，sspA，gtrB，cylG，cyaB，pvdH，fbpC) iron(III，hitC) iron(III，katA，fbpC) iron(III，cylR2，farA，farB，fbpC) iron(III，hitC) iron(III，IlpA，adsA，srtC，pvdH，yhxB/manB，bprB，iap/cwhA，iap/cwhA，allS，flmH，algU，hlyA，Cj1436c，cylI，hlyA，pdgA，msbA，msbA，chuW，basC，cylA) ABC (ATP-binding cassette，prsA2，fbpC) iron(III，isdG，esaA，lplA1，chuY，pchD，cylA，bauB，pchD，cheV3，flmH，sipA，lbtC，phzH，tagAB-5，tlpB，pvdH，cylI，fbpC) iron(III，hitC) iron(III，hitC) iron(III，fbpC) iron(III，bapC，flmH，cylA) ABC (ATP-binding cassette，farB，galE，hddA，mgtC，rhlB，hlyB，bopD，ami，gtrB，mucD，farB，fbpC) iron(III，iap/cwhA，hitC) iron(III，cba，lgtF，cylA，fepC，sfaX，fliA，fleS/flrB，pilS，sfaX，motD，motA，clpE，flmH，iap/cwhA，msrA/B(pilB)，mgtB，bopD，acfB，gnd，fleS/flrB，Cj1437c，cheV，cylG，ptxR，bplB，fbpC) iron(III，sipA，bauE，pilS，allB，clbG，wcbR，clbI，wcbR，wcbR，wcbR，clbI，wcbR，clbP，pdgA，aur，kdtB，vpdC，mucD，wecA，cpsG，fliA，fliA，fbpC) iron(III，lspA，allB，cysC，fbpA，mgtB，wbkC，pppA，ppkA，clbG，cylG，acpXL，flhF，flhF，flhB，fimB，clpE，flgB，flgC，fliE，fliF，fliG，cdsL，cdsN，flgD，flgG，fliL，fliM，fliN，cheY，fliP，fliQ，fliR，flhB，flhA，flhF，fleN，cheB，cheA，cheW-2，cheD，fliA，cpsA，cpsB，mucP，CBU_1434，pdgA，essC，cylG，clbP，wcbT，clbB，clbB，clbB，pvdL，wcbR，wcbR，pvdI，wcbR，cylA，eccA3，wcbR，cheB-2，msrA/B(pilB)，wcbR，cylG，bopD，phtA，fbpC) iron(III，phoR，phoP，pvdI，pvdI，pvdD，clbG，cylG，wcbT，pvdH，hasC，entE，clbF，pvdI，pvdL，pvdI，pvdI，pvdL，aut，fimE，fimE，dep/capD，allS，wcbR，allS，cylG，exsA，iap/cwhA，lap，sodB，sodCI，iap/cwhA，iroB，lpxE，pvdH，cpsJ，cap8E，pseB，msrA/B(pilB)，msrA/B(pilB)，fleQ/flrC，AHA_3493，per，lsgC，Cj1437c，amoA，cheR-3，ebp，algU，phoR，bfmR，msrA/B(pilB)，sipA，lirB，fliA，fimE，flmH，clbI，wcbR，clbI，wcbR，wcbR，clbI，wcbR，cylG，pvdL，clbB，allS，gnd，fbpC) iron(III，pebA，lafK，icl) Isocitrate lyase Icl (isocitrase，clbF，clbD，cheB，recN，vpdC，ideR，lplA1，cylF，pilE，mshG) MSHA (mannose-sensitive hemagglutinin，xcpR，hlyA，hitC) iron(III，hitC) iron(III，sodB，CBU_2076，fliA，CBU_1594，lpg2936，chuW，pce，pdgA，bfmR，coxH2，icaR，aut，fimE，fbpC) iron(III，pebA，relA) Probable GTP pyrophosphokinase RelA (ATP:GTP 3'-pyrophosphotransferase) (PPGPP synthetase I) ((P)PPGPP synthetase，iap/cwhA，pvdN，flhG，pilD，pvdH，vasH/clpV，clpC，orfM，sfaX，csgD，pchD，algR，algZ，phoR，phoP，cylG，entE，pvdN，icaR，pchD，motB，pomA2，bopD，kdsA，essC，wzt，bopD，hitC) iron(III，fleS/flrB，bfmR，fbpC) iron(III，hitC) iron(III，farB，ricA，phzH，fbpC) iron(III，napA，luxS，entE，pchA，lap，bslA/yuaB，aut，aut，fimE，cpsO，fimB，bplA，acfB，acfB，acfB，acfB，hlyA，lap，lap，Cj1437c，rhlA，Cj1437c，fleS/flrB，lbtB，fleS/flrB，fleR/flrC，chuV，bprB，phzD1，entA，esaA，essC，essB，esaB，esxA，mbtH，pvdD，entB，pchD，amoA，entA，iroE，wzt，flmH，adsA，allB，lbtC，allC，fbpC) iron(III，pvdN，rtxB，IlpA，fbpC) iron(III，clbF，clbD，sfaX，farB，allS，fepC，cylG，napA，mucD，bfmR，phoR，bprB，fepC，chuU，shuT，flmH，CBU_1789，phoR，phoP，fepC，shuU，fepD，bauB，bprB，cylA) ABC (ATP-binding cassette，mgtB，mgtB，bplA，hitC) iron(III，allS，cylG，bfmS，bfmR，cylA) ABC (ATP-binding cassette，fbpC) iron(III，fbpC) iron(III，per，rffG，bplF，tviB，scpA，lbtC，bopD，allS，bopD，rpoN，per，neuD，cap8M，kfoC，cpsE，cps4H，wbcG，wbbO，cap8D，cap8B，cps4C，bopD，clpP，allS，lbtC，iap/cwhA，phoR，bfmR，msbA，wcsT，cylB，farB，pvdG，fbpC) iron(III，fliS，fliD，flaB，fliW，flgL，lfgK，bprB，cps4A，wecA，kfiC，algD，waaG，cap8M，cpsA，cps4I，hasC，cpsJ，cpsK，cpsC，cpsD，rfaE，cpsC，lytB，kfiC，cpsA，iap/cwhA，capA，capC，capB，bopD，rfaE，fbpC) iron(III，ptxR，dep/capD，algD，cap8C，cap8B，cap8A，fabZ，flgG，flgF，farB，phzD1，plcD，ureB，ureA，ureA，yscN，yscN，wzb，escN，phoP，msbA，plcD，prfA，cylR2，hitC) iron(III，allS，flmH，Cj1437c，cylG，lbtC，lbtC，phoR，bfmR，bauE，cap8F，rffG，rffG，hasC，flmD，neuB，bplF，Cj1434c，cpsJ，scpA，gtcA，allS，fbpC) iron(III，mycP3，lgtC，relA) Probable GTP pyrophosphokinase RelA (ATP:GTP 3'-pyrophosphotransferase) (PPGPP synthetase I) ((P)PPGPP synthetase，pvdI，algI，fepD，fepG，bauB，katA，bsh，hlyB，ybtQ，icaB) N-deacetylase, involved in polysaccharide intercellular adhesin(PIA，fbpC) iron(III，galE，katA，bauB，fbpC) iron(III，fleS/flrB，bfmR，bplA，cylG，bprB，coxFIC1，pvdH，lafK，bauE，phoR，algW，phoR，bfmR，lsgC，cps4H，ptxR，ptxR，cylG，flpF

Supplementary Table 5 The genes involved in the synthesis of resistance inducers and plant growth promotion in JT-3 and JD-3.

Trait	JT-3			JD-3		
	**Gene_ID**	**Function**	**Gene name**	**Gene_ID**	**Function**	**Gene name**
IAA Production	GE000010	Probable tryptophan transport protein	*trpP*	GE000989	Probable tryptophan transport protein	*trpP*
	GE000129	Tryptophan--tRNA ligase	*trpS*	GE001110	Tryptophan--tRNA ligase	*trpS*
	GE001078	Tryptophan synthase alpha chain	*trpA*	GE002129	Tryptophan synthase alpha chain	*trpA*
	GE001079	Tryptophan synthase beta chain	*trpB*	GE002130	Tryptophan synthase beta chain	*trpB*
	GE001080	N-(5'-phosphoribosyl)anthranilate isomerase	*trpF*	GE002131	N-(5'-phosphoribosyl)anthranilate isomerase	*trpF*
	GE001081	Indole-3-glycerol phosphate synthase	*trpC*	GE002132	Indole-3-glycerol phosphate synthase	*trpC*
	GE001082	Anthranilate phosphoribosyltransferase	*trpD*	GE002133	Anthranilate phosphoribosyltransferase	*trpD*
	GE001083	Anthranilate synthase component 1	*trpE*	GE002134	Anthranilate synthase component 1	*trpE*
Phosphate solubilization	GE001327	Phosphate import ATP-binding protein PstB 1	*pstB1*	GE002376	Phosphate import ATP-binding protein PstB 1	*pstB1*
	GE001328	Phosphate import ATP-binding protein PstB 2	*pstB2*	GE002377	Phosphate import ATP-binding protein PstB 2	*pstB2*
	GE001329	Phosphate transport system permease protein PstA 2	*pstA2*	GE002378	Phosphate transport system permease protein PstA 2	*pstA2*
	GE001330	Phosphate transport system permease protein PstC	*pstC*	GE002379	Phosphate transport system permease protein PstC	*pstC*
	GE001331	Phosphate-binding protein PstS	*pstS*	GE002380	Phosphate-binding protein PstS	*pstS*
Nitrogen fixation	GE001503	Putative cysteine desulfurase NifS	*nifS*	GE002610	Putative cysteine desulfurase NifS	*nifS*
	GE001921	Nitrogen fixation protein NifU (Fragment)	*nifU*	GE003128	Nitrogen fixation protein NifU (Fragment)	*nifU*
	GE000738	Glutamine synthetase	*glnA*	GE001722	Glutamine synthetase	*glnA*
	GE001959	Zinc-dependent sulfurtransferase SufU	*sufU*	GE003168	Zinc-dependent sulfurtransferase SufU	*sufU*
Hydrolase	GE000808	Endoglucanase	*bglC*	GE001802	Endoglucanase	*bglC*
	GE002070	Carboxylesterase	*est*	GE003281	Carboxylesterase	*est*
	GE003015	Lipase EstA	*estA*	GE000281	Lipase EstA	*estA*
	GE000718	Serine protease AprX	*aprX*	GE000416	Spore germination lipase LipC	*lipC*
	GE001109	Protease PrsW	*prsW*	GE001701	Serine protease AprX	*aprX*

	GE003042	Alpha-amylase	*amyE*	GE002160	Protease PrsW	*prsW*
				GE000308	Alpha-amylase	*amyE*
Chitinase activity	GE002780	Cortical fragment-lytic enzyme	*sleL*	GE000045	hydrolase, family 18	
	GE000316	Putative sporulation-specific glycosylase YdhD	*ydhD*	GE001350	hydrolase, family 18	
				GE000045	Cortical fragment-lytic enzyme	*sleL*
				GE001301	Putative sporulation-specific glycosylase YdhD	*ydhD*
Biofilm	GE001258	Stage 0 sporulation protein A	*spo0A*	GE002308	Stage 0 sporulation protein A	*spo0A*
	GE001868	Degradation enzyme regulation protein DegQ	*degQ*	GE002346	HTH-type transcriptional regulator SinR	*sinR*
	GE001297	Major biofilm matrix component	*tasA*	GE002347	Major biofilm matrix component	*tasA*
	GE001798	Biofilm-surface layer protein A	*bslA*	GE002349	TasA anchoring/assembly protein	*tapA*
	GE002482	Probable biofilm-surface layer protein B	*bslB*	GE002913	Biofilm-surface layer protein A	*bslA*
	GE003520	Putative membrane-bound acyltransferase YfiQ	*yfiQ*	GE003075	Degradation enzyme regulation protein DegQ	*degQ*
	GE001296	HTH-type transcriptional regulator SinR	*sinR*	GE003362	HTH-type transcriptional regulator SlrR	*slrR*
	GE002149	HTH-type transcriptional regulator SlrR	*slrR*	GE000821	Putative membrane-bound acyltransferase YfiQ	*yfiQ*
